# The Potential of Algal Biotechnology to Produce Antiviral Compounds and Biopharmaceuticals

**DOI:** 10.3390/molecules25184049

**Published:** 2020-09-04

**Authors:** Sergio Rosales-Mendoza, Ileana García-Silva, Omar González-Ortega, José M. Sandoval-Vargas, Ashwini Malla, Sornkanok Vimolmangkang

**Affiliations:** 1 Laboratorio de Biofarmacéuticos Recombinantes, Facultad de Ciencias Químicas, Universidad Autónoma de San Luis Potosí, Av. Dr. Manuel Nava 6, San Luis Potosí 78210, Mexico; a196037@alumnos.uaslp.mx (I.G.-S.); omar.gonzalez@uaslp.mx (O.G.-O.); jsandovalv1702@alumno.ipn.mx (J.M.S.-V.); 2 Sección de Biotecnología, Centro de Investigación en Ciencias de la Salud y Biomedicina, Universidad Autónoma de San Luis Potosí, Av. Sierra Leona 550, Lomas 2^a^. Sección, San Luis Potosí 78210, Mexico; 3 Department of Pharmacognosy and Pharmaceutical Botany, Faculty of Pharmaceutical Sciences, Chulalongkorn University, Bangkok 10330, Thailand; malla164_ashwini@yahoo.co.in; 4 Research Unit for Plant-Produced Pharmaceuticals, Chulalongkorn University, Bangkok 10330, Thailand

**Keywords:** recombinant antigen, monoclonal antibody, *Chlamydomonas reinhardtii*, transplastomic, COVID-19, SARS-CoV-2, MERS-CoV

## Abstract

The emergence of the Coronavirus Disease 2019 (COVID-19) caused by the SARS-CoV-2 virus has led to an unprecedented pandemic, which demands urgent development of antiviral drugs and antibodies; as well as prophylactic approaches, namely vaccines. Algae biotechnology has much to offer in this scenario given the diversity of such organisms, which are a valuable source of antiviral and anti-inflammatory compounds that can also be used to produce vaccines and antibodies. Antivirals with possible activity against SARS-CoV-2 are summarized, based on previously reported activity against Coronaviruses or other enveloped or respiratory viruses. Moreover, the potential of algae-derived anti-inflammatory compounds to treat severe cases of COVID-19 is contemplated. The scenario of producing biopharmaceuticals in recombinant algae is presented and the cases of algae-made vaccines targeting viral diseases is highlighted as valuable references for the development of anti-SARS-CoV-2 vaccines. Successful cases in the production of functional antibodies are described. Perspectives on how specific algae species and genetic engineering techniques can be applied for the production of anti-viral compounds antibodies and vaccines against SARS-CoV-2 are provided.

## 1. Introduction

Coronaviruses are enveloped viruses having single-stranded, positive sense RNA genome carrying the spike protein on their surface that mediate virus entry into the target cell [1]. The emerging Coronavirus Disease 2019 (COVID-19), caused by the Severe Acute Respiratory Syndrome Coronavirus 2 (SARS-CoV-2), possesses high transmissibility and has led to a worldwide public health crisis. Following its first description in Wuhan, China; SARS-CoV-2 has rapidly spread around the world. COVID-19 was declared a pandemic on March 2020 [2] and by the mid of August over 23 million people were infected by SARS-CoV-2 with more than 800,000 deaths registered. COVID-19 symptoms range from mild flu-like illness to potentially lethal acute respiratory distress syndrome or fulminant pneumonia, the latter considered as the critical/dominant clinical manifestation [3]. SARS-CoV-2 is related to SARS-CoV-1 [4], that gained attention/prominence after the SARS outbreaks in 2003, and the Middle East Respiratory Syndrome virus (MERS-CoV) that emerged in 2012 [5]. 

The impact of the COVID-19 pandemic in economic, loss of production/jobs, commercial/trade restrictions, and large investment in control and prevention, and health terms (morbidity and mortality) make finding specific treatments an urgent goal. By now strategies comprising antivirals and corticosteroid therapy, together with mechanical respiratory support, are considered the front-line treatment [3]. Since vaccines to prevent COVID-19 are unavailable, there is an urgent need to develop antiviral drugs, anti-inflammatory drugs, and antibodies to fight against this disease in the short term; while accelerating the development of vaccines that would be the ideal strategy to fight against this disease in the midterm [6]. The most advanced vaccine candidates are already under clinical evaluation and include formulations based on mRNA (Moderna, Cambridge, MA, USA), adenoviral vectors (CanSino Biologicals, Tianjin, China; and Oxford University/Astra Zeneca, Cambridge, UK), and INO-4800 (Inovio, Plymouth Meeting. PA, USA) [7].

Microalgae and cyanobacteria involve a diverse group of unicellular organisms found in aquatic (fresh- and sea-water) and terrestrial environments [8]. They are capable of growing either photoautotrophically or heterotrophically depending on the type of available carbon source; making their cultivation potentially simple and cost-effective [9]. Micro and macroalgae and cyanobacteria have gained attention due to their unique metabolic pathways, whose products could be a source of commercially valuable products such as carotenoids, polyunsaturated fatty acids, proteins, phycobiliproteins, and polysaccharides [10]. Many of these compounds have antiviral and anti-inflammatory activities with a potential application in the development of drugs and treatments against COVID-19. The advances achieved during the last decades in genetic engineering of algae have paved the way for the implementation of bioprocesses based on algae strains with improved traits for an efficient production of native or recombinant products [11,12,13,14,15]. This is especially useful for the case of target compounds produced in trace amounts or those not being naturally produced by the algae species [12,16]. Genetic engineering either by nuclear or organelle expression has been demonstrated for algae species [17]. When compared to plants (also attractive hosts to produce bioactive metabolites and biopharmaceuticals), microalgae expression systems ranging from industrial to commercial applications offer considerable advantages that include high scalability with better growing rates (5–10 fold higher), low production costs, and increased biomass culture with simple mineral requirements. Moreover, wastewater or water unsuitable for human consumption can be used for algae growth [18].

An additional advantage for algae cultures corresponds to the lack of competition for agricultural land; making them a sustainable approach as excellent “cellular factories” to produce high-value compounds [19], while reducing the carbon dioxide levels generated by anthropogenic//human activities [20]. Microalgae strains are commonly grown for the production of functional foods and aquaculture products given their contents of functional and nutritional compounds [21,22]. However, the large scale industrial exploitation of micro- and macroalgae-derived compounds is largely limited to phycocolloids (carrageenan, agars, and alginates) for their gelation, emulsifying, and water-holding capacities; and biochemicals (carbohydrates, lipids, minerals, pigments, and low molecular weight compounds). These compounds are mainly employed as bulk or specialty commodities in foods, food additives, nutraceuticals, feed industries [21], and biofuels production [23]. The groups of algal species exploited by the industry include *Dunaliella salina* (β-carotene), *Haematococcus lacustris* (astaxanthin), *Chondrus* and *Eucheuma* (carrageenans), *Sargassum sinicola* (alginates), *Undaria pinnatifida* (fucoxanthin), and *Chlorella vulgaris* (fatty acids and triglycerides) [24,25,26,27,28].

**Figure 1:**
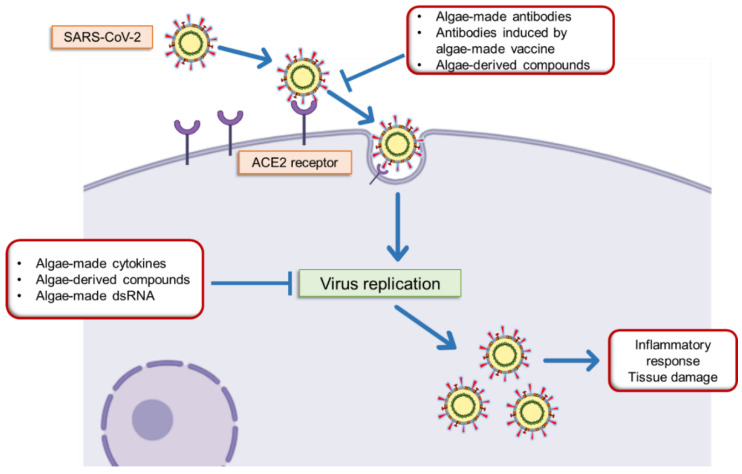
Simplified view of the SARS-CoV-2 pathogenic mechanisms and possible algae-based products to fight against it. The SARS-CoV-2 access the cells at the airway mucosa by targeting the ACE2 receptor. Upon cell entry, viral replication takes place and induces tissue damage that might result in a severe inflammatory response and systemic spread, which can cause death; especially in patients suffering of co-morbidities. Microalgae can be exploited in several directions as sources of drugs and biologicals in the fight against SARS-CoV-2 infection. Algae-derived compounds such as lectins and polysaccharides have known ability to block the entry or replication of enveloped viruses. Through genetic engineering; algae can lead to the development of low-cost production platforms for the manufacture of vaccines, monoclonal antibodies, and cytokines; all of them being key biopharmaceuticals in the prevention or treatment of COVID-19.

The present review provides an outlook on how algae biotechnology can be exploited to fight SARS-CoV-2 at different levels through the production of antiviral and anti-inflammatory compounds, recombinant vaccines, monoclonal antibodies, and cytokines (Figure 1).

## 2. Algae-Derived Antiviral Compounds

Although a significant number of antiretroviral drugs are available in the market [29], the development of new therapies and prophylactic treatments for viral infections is still an urgent goal; given the rapid evolution of viruses. Algae are interesting hosts for the discovery and production of bioactive compounds; many species are Generally Recognized as Safe (GRAS) organisms due to the absence of human-related endotoxins, viruses, or pathogens [30]. The bioactive compounds produced in algae [31,32] include fucoidans [33], lectins [34,35], polysaccharides [36], and proteins [37]. 

### 2.1. Pigments

Algae and cyanobacterial pigments are associated to light harvesting, CO_2_ fixation, cell protection from excessive irradiation, and ultimately giving the characteristic pigmentation to the culture [38]. The wide range of pigments that can be produced by microalgae includes carotenoids, chlorophyll, and phycobiliproteins; with many of them having relevance in the food and drug industries [39]. Microalgal carotenoids are the most relevant compounds in terms of commercial exploitation and are essential for the growth of algae since these act as protective agents from reactive oxygen species and high irradiation [40]. β-carotene produced in *D. salina* [41] and astaxanthin extracted from *H. lacustris* [42] are important carotenoids. Talukdar et al. [43], proposed the use of astaxanthin (nASX) as adjunctive supplement given its potential for alleviating cytokine storm, acute lung injury, and acute respiratory syndrome [44]. However, the beneficial or supportive role in alleviating COVID-19 symptoms must be demonstrated.

Phycobilins are the most studied pigments for their bioactive properties and are only produced by cyanobacteria such as *Nostoc* sp., *Oscillatoria* sp., *Spirulina* sp., and *Anabaena* sp. Phycobilins are unique photosynthetic pigments since these are bound to water-soluble proteins, namely phycobiliproteins; conferring them bioactive effects [45]. Phycobiliproteins are used in photodynamic therapy (PDT) as chemical-pigment tags [46] and pharmaceutical applications due to their antioxidant and anti-inflammatory activities [47].

Phycoerythrin is a red protein pigment that is abundant in Rhodophyta and cyanobacteria with antitumor and anti-ageing properties [48]; it has also been reported as an anti-inflammatory compound [49]. Fucoxanthin, a xanthophyll-like carotenoid, has also shown many biological properties that include anti-inflammatory effects [50,51]. 

Zeaxanthin and lutein produced by *D. salina*, *Chlorella protothecoides*, and *Spirulina maxima* exerted anti-inflammatory action against endotoxin-induced uveitis (EIU) [52]. Violaxanthin; an orange colored natural xanthophyll found in *Chlorella ellipsoidea* [53] and *Dunaliella tertiolecta* [54] acts as a potential anti-inflammatory agent against many infections by suppressing the formation of NO and PGE2 in RAW 264.7 cells. 

### 2.2. Polyphenols 

As secondary metabolites, polyphenolic molecules include phenolic acids, flavonoids, isoflavonoids, stilbenes, lignans, and phenolic polymers [55]. Similar to other bioactive molecules, the content and composition from algae polyphenols are species-dependent [56]. These molecules display a wide range of bioactivities including antioxidant, anti-inflammatory, anti-cancer, antiallergic, antidiabetic, anti-aging, and antimicrobial properties [55]. Polyphenols are produced by most plants and algae with demonstrated biological properties [43,44]. As for their antioxidant capacity, some studies establish that ingestion of food with high antioxidant levels might result in protection against oxidative stress [57].

Polyphenolic compounds have demonstrated antiviral activity against HIV [58], Herpes Virus (HV) [59,60], and Measles Virus (MV) [61]. Among them, phlorotannins biosynthesized via the acetate malonate pathway [62] comprise a whole spectrum of molecules produced by brown seaweed [63] with anti-allergic [64], antioxidant [65], and photoprotective [66] properties; with noticeable bioactivity in virus-related studies that include the Influenza virus [67,68], HIV [69], and Hepatitis Virus [70]. Morán et al. [71] tested the in vitro antiviral activity of polyphenols from five Mexican seaweeds [62] against the Measles Virus (MeV). They assessed the combined antiviral effect of polyphenols and sulfated polysaccharides isolated from the seaweeds with a synthetic nucleoside to discover new antiviral drug candidates that could help controlling viral diseases [71]. Brown algae from the *Dictyotaceae* family produce various secondary metabolites, especially diterpenes. Based on the cyclization of the geranyl-geraniol precursor, diterpenes are categorized into various groups such as dolabellanes, sesquiterpenes, and xenicanes. The derivatives of diterpenes isolated from the red macroalgal species *Dictyota pfaffii* and *Dictyota menstrualis* exhibited anti-HIV activity with low toxicity, and thus are considered promising candidates for drug development [72]. Fucosterol, abundant in brown algae (*Eisenia bicyclis*, *Fucus vesiculosus*, and *Turbinaria conoides*), is also widely studied for its in vitro properties and could be an efficient therapeutic agent for various health problems [73,74].

The metabolic diversity of algae offers attractive candidates to be exploited in the medical field, including the development of treatments against COVID-19 [75]. An important niche for this field consists in applying genetic engineering to improve the production of polyphenols in algae as this field is restricted to scarce studies based on UV-stress using *Scenedesmus quadricauda* [76]. The perspectives section of this review provides new paths to guide the reader on possible genetic engineering developments to innovate this field. 

### 2.3. Lectins

Lectins are proteins that reversibly bind to certain mono and oligosaccharides; lacking of catalytic activity [77]. Lectins are ubiquitous in nature and have been identified in prokaryotic and eukaryotic species. In the case of algae, lectins have been proposed for several applications that include the development of antiviral therapies [78,79]; given the known anti-viral activity of such compounds, which is attributed to glycocalyx depletion at the surface of enveloped viruses [80]. 

Amongst the relevant described lectins; cyanovirin-N (CVN), isolated from the cyanobacteria *Nostoc ellipsosporum* [81], has been demonstrated to inhibit viral entry for the cases of HIV [82], Ebola [83], and influenza virus [84]. Other lectins with demonstrated anti-HIV activity are scytovirin (SVN) from *Scytonema varium* [37] and agglutinin from *Oscillatoria agardhii* [85]. A prominent example of algae-derived lectins is griffithsin, which is covered in detail in the following section.

#### Griffithsin: A Promising Algae-Derived Polypeptide with Anti-SARS-CoV-1 and MERS-CoV Activity

Griffithsin (GRFT) is a 121 amino acid lectin produced by the red macroalga *Griffithsia* sp., that possesses potent (EC50 in the picomolar range), broad-spectrum antiviral activity with null toxicity [86]. The antiviral activity of GRFT is associated to the formation of homodimeric complexes displaying three carbohydrate-binding domains per monomer, which target high-mannose arrays at the surface of pathogenic enveloped viruses; such as the human immunodeficiency virus (HIV) and the Severe acute respiratory syndrome (SARS-CoV-1) and Middle East respiratory syndrome coronaviruses (MERS-CoV). 

GRFT has been primarily investigated as antiviral agent against HIV-1. Remarkably, both the native and recombinant GRFT (produced in *Escherichia coli*) displayed cytoprotective activity against HIV-1 at sub-nanomolar concentrations [34]. One of the key findings regarding the mechanisms of action of GRFT came when it was proven that it impeded the interaction between gp120 and CD4 receptor-expressing cells, an effect dependent on the glycans present in gp120 that block viral fusion. It was then deduced that the high antiviral potency of GRFT derived from multivalent interaction via its three carbohydrate-binding domains that target high-mannose type oligosaccharides [87]. Moreover, tyrosine residues (such as Tyr28, Tyr68, and Tyr110) are also involved [88]. The antiviral mechanisms of GRFT have been characterized for the case of HIV-1. By using monoclonal antibodies (mAbs) targeting HIV it was shown that GRFT enhanced the interaction between gp120 and 48d mAb, which targets a CD4-induced epitope [89]. This suggested that the binding of GRFT to gp120 leads to the display of the CD4-binding site. A binding competition between GRFT and gp120 for the dendritic cell-specific intercellular adhesion molecule-3-grabbing nonintegrin (DC-SIGN) has also been proposed [90]. GRFT induces a partial blockade of gp120 binding to human DC-SIGN; therefore, inhibiting HIV transfer [91]. 

Importantly, GRFT specifically binds to the SARS-CoV-1 spike (S) glycoprotein and inhibits viral entry in a concentration-dependent manner [92]. In vitro assays performed with Vero 76 cells have demonstrated the anti-SARS-CoV-1 activity of GRFT using four distinct strains. Moreover, an in vivo evaluation confirmed a potential inhibition of SARS-CoV-1. BALB/c mice were intranasally administered with GRFT and challenged 4 h later with a mice-adapted MA15 SARS-CoV-1 strain; they received 2 daily GRFT doses during the following 4 days. Mice subjected to the treatment showed neither mortality nor weight loss; displaying reduction in lung tissue virus titers and viral antigens. Inhibition of MERS-CoV by GRFT has also been assessed in vitro [93]. The effects of GRFT on cell viability and the inhibitory activity on MERS-CoV infectivity were evaluated in Huh-7, MRC-5, and Vero-81 cells; observing no significant cytotoxicity with substantial decrease in the MERS-CoV infectivity in a dose-dependent manner. Furthermore, MERS-CoV pseudotyped virions were used to infect Huh-7 cells and the influence of GRFT on the MERS-CoV S protein-mediated entry was determined; observing a dose-dependent inhibition. In addition, through a competition assay, it was shown that GRFT interacts with mannoses from the MERS-CoV S envelope impairing their function during entry. 

These precedents highlight the antiviral potential of GRFT against coronaviruses, while not exerting cytotoxicity. The pharmacokinetic profile of GRFT upon administration by different routes (oral, intravenous, and subcutaneous) was evaluated in Sprague Dawley rats; revealing that therapeutic GRFT levels were sustained up to 96 h upon intravenous and subcutaneous administration. Although GRFT was not detected in serum following oral administration, it was detected in feces 8 h post-administration. Even though the optimal therapeutic concentration should be determined for each species, the results suggest that GRFT can be used to treat systemic and enteric viral infections [94]. The safety of GRFT as potential systemic antiviral treatment was evaluated in BALB/c mice and Hartley guinea pigs subjected to daily subcutaneous administration-based schemes [95]. GRFT was systemically accumulated at relevant therapeutic concentrations, which were tolerated with minimal toxicity in treated animals with single and chronical subcutaneous administration; moreover, serum of GRFT-treated animals showed antiviral activity against HIV-1. Furthermore, in human peripheral blood mononuclear cells (PBMCs), GRFT did not result in major alteration of the secretion of inflammatory cytokines and chemokines without significant effects in cell viability or levels of T-cell activation markers; in addition to maintaining its activity once bound to PBMCs [96]. 

GRFT exhibits no cytotoxicity when assessed in several cell types at concentrations up to 500 nM [97]. In other studies, the toxicological profile of GRFT was determined in mice under acute or chronic treatments based on subcutaneous and intravaginal administration. GRFT caused no significant cell death, mitogenicity, and activation or cytokine release in PBMCs of mice [98]. Furthermore, in vivo studies showed that GRFT was not inherently toxic in mice. Evaluations using cervical explants and an in vivo rabbit vaginal irritation model revealed that GRFT did not provoke irritation or inflammation. Moreover, assays performed with human lymphocytes revealed that GRFT has no mitogenic activity [99]. There are two ongoing clinical studies evaluating the potential toxicity of GRFT [100,101]. 

Different efforts have been reported pursuing the development of a practical GRFT-based antiviral treatment; especially considering that large-scale production is an important requirement for clinical application. Recombinant GRFT production systems and their optimization have been reported for the following hosts: *E. coli*, *Nicotiana benthamiana*, *Lactobacillus rhamnosus*, and rice endosperm [102,103,104,105]. 

Despite that GRFT has shown resistance to several proteases, some authors have focused on the development of GRFT delivery systems based on poly(lactide-co-glycolide) (PLGA) nanoparticles [106] and electrospun fibers [107], which are intended to result in controlled delivery [108]. Core-shell PLGA nanoparticles (180–200 nm) successfully encapsulated 45% of the initial GFRT and, in combination with the antiretroviral drug dapivirine, showed biphasic and sustained release maintaining bioactivity in a cell-based assay [106]. Moreover, fibers prepared with polyethylene oxide (PEO), polyvinyl alcohol (PVA), and polyvinylpyrrolidone (PVP) have been designed for rapid-release of GRFT and evaluated against HIV-1 and the herpes simplex virus 2 (HSV-2) in vitro and in vivo [109]. High levels of GRFT incorporation in all formulations and potent protection in a murine model infection were achieved without increasing cytokine levels or histological damage in vaginal lavages and reproductive tissues; demonstrating the safety of the polymeric fibers. Therefore, GRFT is a remarkable antiviral agent and it is imperative to assess its potential against SARS-CoV-2. 

### 2.4. Polysaccharides

Polysaccharides are mostly found in algae in the form of heteropolymers [110] with *Gyrodinium impudicum* and *C. vulgaris* as sole algal species producing homopolymer polysaccharides [111]. Sulfated polysaccharides (SPs) are common in algae and these polyanionic molecules have been investigated for the treatment of a wide spectrum of viral infections [112,113]; specifically for HIV, the Herpes Simplex Virus (HSV), African swine fever virus (ASFV), and influenza A virus (Flu-A). Among several kinds of algal polysaccharides, carrageenans are the most studied and considered safe for human use [114,115]. Other algal polysaccharides that include fucans and ulvans have been characterized and considered attractive for antiviral drug development. 

Carrageenans are sulfated polysaccharides found in red algae (Rhodophyta) including the genera *Chondrus*, *Gigartina*, *Hypnea*, and *Eucheuma*; wherein they have a similar structural role to cellulose in plants [116]. Carrageenans can be divided into six groups depending on the chemical structure: iota (ι)-, kappa (κ)-, lambda (λ)-, mu (μ)-, nu (ν)- and theta (θ)-forms, which naturally occur as mixtures in the individual alga species. Carrageenans ι, κ, and λ are the most studied for their antiviral activities. κ-carrageenan and ι-carrageenan have similar ester sulfate content and number of anhydrogalactose units, while λ-carrageenan has higher sulfate content without anhydrogalactose content. The proven antiviral mechanisms of carrageenans include inhibition of viral attachment and uncoating as well as transcription, replication and immune function modulation. Viral attachment blocking is influenced by the size of carrageenans and sulfation degree [117]. However, low molecular weight (LMW) derivatives of carrageenans also displayed antiviral effects. LMW carrageenans can occur naturally by degradation or they can be obtained by free radical depolymerization, mild acid hydrolysis, or enzymatic degradation [28,29,30]. The method of depolymerization may affect the antiviral activity. The antiviral activity of LMW derivatives of κ- and κ/β-carrageenans was strongest by mild acid hydrolysis; followed by free radical depolymerization and enzymatic degradation [118]. LMW carrageenans can penetrate the host cell and inhibit viral replication. For example, κ-carrageenan oligosaccharides (KCO) showed this effect on the influenza A virus [119,120].

ι-carrageenans not only inhibit viral attachment, but also viral internalization. ι-carrageenans blocked the attachment of HSV and the Dengue virus [121,122]. Viral duplication of rhinovirus (HRV) was blocked by ι-carrageenans [123]. ι-carrageenans significantly reduced viral replication and increased survival of cells infected by the influenza virus H1N1 strain [124]. Due to the low solubility and inhibition of viral attachment of carrageenans, ι-carrageenans were formulated as nasal spray and clinically approved for common cold in Europe. In clinical studies, the nasal spray significantly reduced the symptoms of the common cold, decreased viral load, and reduced inflammation in patients [125,126,127]. Koenighofer et al. [128] reported that the carrageenan nasal spray decreased the duration of common cold disease in patients. The addition of zanamivir (an antiviral drug) to the carrageenan nasal spray was synergistically active against the Influenza A virus [129]. The combination of ι-carrageenans and LMW oligosaccharides increased the antiviral efficiency [130]. In addition, there are numerous studies on the antiviral effects of λ- and κ-carrageenans. The viral attachment was blocked by λ-carrageenans for several human and animal viruses including the herpes simplex virus 1 and 2 (HSV-1 and HSV-2), equid herpesvirus 3 (EHV3), bovine herpes virus 1 (BoHV-1), suid herpes virus 1 (SuHV-1), and feline herpes virus 1 (FeHV-1) [131,132,133]. Shao et al. [134] found that κ-carrageenans can block viral attachment of A/Swine/Shandong/731/2009 H1N1 (SW731). 

Fucans are high molecular weight sulfated polysaccharides found in the cell walls of brown algae; they are classified in three major groups: glycuronogalactofucans, fucoidans, and xylofucoglycuronans. Fucose is attached to the central backbone, mainly by glycosidic linkages, forming branching points every 2–3 fucose residues within the chain [135]. Fucoidans are the most studied for their antiviral activity. Fucoidan derived from the extracellular matrix of several brown algae has a high content of fucose; which is the case of the following species: *Cladosiphon okamuranus* (mozuku), *Saccharina japonica* (komby), *Sphaerotrichia divaricata* (limu moui), *F. vesiculosus* (bladder wrack), *U. pinnatifida* (wakame), *Sargassum fusiforme* (hijiki), and *Holothuroidea* (sea cucumber). Fucoidans from several brown algae were reported for their anti-HIV activity. Fucan A and B from *Spatoglossum schroederi* and *Dictyota mertensii* can inhibit viral transcription and replication of HIV [136,137]. Other fucans from *Lobophora variegata* and *F. vesiculosus* showed strong inhibitory effect on the reverse transcriptase enzyme of HIV-1. Fucoidans from three brown algae (*Sargassum mcclurei*, *Sargassum polycystum*, and *Turbinaria ornata*) inhibited the HIV-1 viral entry point on the host cell [138]. Fucoidans have been tested for anti-influenza A virus (IAV) activity in vitro and in vivo. Akamatsu et al. [139] evaluated MC26, which is a fucose polysaccharide from the marine brown alga *Sargassum piluliferum* and possesses superior anti-influenza virus effects with low cytotoxicity (in vivo and in vitro) respect to known active compounds such as amantadine and MC24 from *T. ornata*. Fucoidan from *U. pinnatifida* has anti-HSV activity [140] and anti-IAV activity in vitro and in mice with normal and compromised immunity [141]. Jiao et al. [142] screened the antiviral activity against the influenza A/PR/8/34 (H1N1) virus; the highest anti-influenza activity was found for fucoidans from *F. vesiculosus* and *Ascophyllum nodosum*. Wang et al. [130] isolated fucoidan from *Kjellmaniella crassifolia* Miyabe and found that it increased the survival rate and lifespan of mice infected with influenza viruses and reduced viral load. Moreover, the most susceptible strain was H1N1 (Ca109) and the antiviral mechanism could be blocking viral penetration; inhibiting the activation of the epidermal growth factor receptor. Nasal and oral administrations of fucoidan are suggested and application at early infection is recommended. Fucoidans from *Macrocystis pyrifera, A. nodosum*, *U. pinnatifida*, and *F. vesiculosus* were found to improve immune function by activation of NK cells, DCs, and T cells. Recently, it was considered that fucoidan could inhibit the release of cytokines from human primary bronchial epithelial cells via the Toll-like receptor 3 (TLR3); suggesting that it could relief bronchial inflammation caused by viral infection when applied locally [143]. Based on results of numerous reports, fucans are promising antiviral agents. Interestingly, a differential structure is observed in fucans from distinct algal species and even in different parts of the seaweed [144,145]. Therefore, sulfated fucans are unique compounds that could lead to the development of bioactive agents.

Ulvan is an algal sulfated polysaccharide found in the cell wall of green macroalgae (Chlorophyta) of the order Ulvales (*Ulva* and *Enteromorpha* sp.). Besides ulvan, other cell wall polysaccharides of the Ulva species are cellulose, xyloglucan, and glucuronan. Ulvans are repeated disaccharide units with sulfated rhamnose residues linked to uronic acids. The antiviral activity of ulvans isolated from *Ulva armoricana*, *Ulva clathrata*, *Enteromorpha compressa (Ulva compressa)*, *Ulva intestinalis, Ulva pertusa*, and *Ulva lactuca* were reported. *U. armoricana* extracts prepared by enzyme-assisted approaches showed antiviral activity against HSV-1 in vitro [146]. The ulvan SU1F1 from *E. compressa* inhibited viral penetration and had virucidal effects on HSV-1 [147]. SPs from *U. intestinalis* had low antiviral activity on the measles virus compared to SPs isolated from the seaweeds *Eisenia arborea* and *Solieria filiformis* [61]. The SPs from *U. pertusa* significantly induced avian influenza virus specific antibodies in vivo [148]. Chiu et al. [149] found that SPs extract from *U. lactuca* showed antiviral activity against the Japanese encephalitis virus. The anti-Newcastle disease viral mechanism of ulvans from *U. clathrata* prevents the cleavage of the viral protein F0 to be mature and the activity was stronger with the combination of fucoidan from *Cladosiphon okamuranus* [150]. Ulvans and fucoidans have the same action mechanism through anti-viral attachment. The synergistic effect can occur with the combined usage. 

Other antiviral polysaccharides from algae are being investigated. Polysaccharides from blue-green algae were reported for their antiviral activities. Calcium spirulan found in *Arthrospira platensis* is an inhibitor of viral replication of HSV-1, human cytomegalovirus, measles virus, mumps virus, IAV, and HIV-1; blocking the virus before penetrating host cells [151]. Nostoflan from *Nostoc flagelliforme* has a viral inhibitory effect on HSV-1, HSV-2, IAV, and human cytomegalovirus [152]. Alginates and laminaran are common polysaccharides found in brown algae. The alginate 911 derivative has inhibitory effect on the viral reverse transcriptase enzyme of HIV; interfering with viral internalization to the host cell and modulating host immunity [153]. Laminaran from kelp blocked HIV replication by inhibiting adsorption and the reverse transcriptase enzyme [154]. The highly sulfated exopolysaccharide p-KG03, which is produced by the marine microalga *G. impudicum*, exerts effects against the Encephalomyocarditis virus in vitro (EC_50_ = 26.9 µg/mL) [155] and also inhibits the influenza A virus infection in vitro [156]. 

Interestingly, the exopolysaccharides (EPS) from *Porphyridium* sp. have shown antiviral activity in vitro and in vivo. EPS from *Porphyridium* sp. are composed of D-xylose, D- and L-galactose, and D-glucose containing glucuronic acid and sulfated groups; several molar ratios of these monosaccharides have been reported [157,158]. Sulfated EPS have shown antiviral activity against the herpes simplex virus types 1 and 2 (HSV-1 and -2) in a concentration-dependent manner in infected cells without cytotoxic effects on Vero cells at concentrations up to 250 µg/mL [159]. Shi-sheng et al. [160] investigated the antiviral effect of EPS against the Respiratory Syncytial Virus (RSV) in the HeLa cell line; observing strong activity against it with little inhibition of cell growth. In addition, EPS from *Porphyridium* sp. have shown antiviral activity against other enveloped viruses such as the viral hemorrhagic septicemia virus (VHSV) and the African swine fever virus (ASFV) [161]; moreover, they have activity against retroviruses such as the murine leukemia virus (MuLV) and murine sarcoma virus (MuSV-124) [162]. The sulfation degree in EPS may be involved in their antiviral activity. EPS produced by a Spanish strain of *Porphyridium cruentum* obtained from sulfated cultures presented higher degree of sulfation and positively influenced antiviral activity [163]. The antiviral activity of EPS is attributed to the inhibition of the binding or internalization of virus into the host cells, suppressing DNA replication and protein synthesis, and to the competence for the glycoprotein-mediated viral attachment [113,164,165].

## 3. Algae-Made Biopharmaceuticals

The notion of using algal species as hosts for the production of recombinant biopharmaceuticals was conceived three decades ago as a system characterized by low cost, rapid production, and enhanced safety; since many species do not produce toxins or carry human pathogens [166]. In addition, the use of algal cells as delivery vehicles could lead to attractive therapies in which no costly purification steps are required. It has been proposed that oral treatments can be implemented using pills or tablets with freeze-dried biomass. However, oral bioavailability for the target biopharmaceutical, especially if it is a systemic target, requires fine optimization [167]. 

The expression of the target biopharmaceutical can be achieved by the established expression approaches at the chloroplast or nucleus, which are mainly optimized for algae model species such as *Chlamydomonas reinhardtii* [168] and *Phaeodactylum tricornutum* [169]. A frequent limitation in this field is associated to low protein yields; as a consequence, many groups have focused on optimizing the expression approaches to overcome this limitation. Some of the improvements achieved in this sense are the generation of mutant strains with better expression of transgenes at the nuclear level [170] and the expansion of signal peptides to allow for an efficient secretion of the recombinant protein [171]. As for the case of chloroplast expression, a series of vectors optimized with specific promoters and UTRs have been described [172,173]. A remarkable example is the use of photorestoration systems in which the use of selectable markers is avoided since the strain carries a mutation that abolish photosynthesis, which is restored upon the foreign DNA insertion that contains the functional gene [174]. Moreover, inducible expression systems have been developed for the chloroplast and constitute a promise for the field (especially when the target biopharmaceutical exerts toxic effects in the algae species used as host) [175] to separate the growth phase from the expression phase as requirement to maximize production.

Viral vectors, e.g., those based on plasmids that lead to the generation of replicons that allow for a massive protein expression, constitute key alternatives in this field. The delivery of such vectors mediated by Agrobacterium is an interesting approach to be explored in green algae; this concept has been successfully applied in other microorganisms, namely the heterokont protist *Schizochytrium* sp. [176]. 

Thus far several biopharmaceuticals have been produced in algae with vaccines as the most explored cases. Some human vaccine candidates have been evaluated at the preclinical level; these candidates include: (i) a vaccine against peanut allergy with the ability to induce immunoprotective effects in a mice peanut-induced anaphylaxis model [177], (ii) a candidate targeting malaria that reduced parasitemia in mice [178], and (iii) a vaccine candidate against the Human papillomavirus with anti-tumoral protection in mice [179]. All these candidates were expressed in the chloroplasts of *C. reinhardtii*. Although not strictly classified as an alga, *Schizochytrium* sp. is a heterokont protist (ancestrally related to photosynthetic heterokonts) that has been used to produce an influenza vaccine candidate; consisting of purified recombinant hemagglutinin that was able to protect mice against a viral challenge (Table 1) [180]. The road ahead in this field requires surpassing the valley of death and achieving the implementation of clinical trials.

Antibodies have also been expressed in microalgae and applied in the fight against cancer and other non-communicable diseases. Among the most advanced models in this category are: an immunotoxin targeting CD22 produced in the chloroplast of *C. reinhardtii*; able to exert cytotoxic effects on B-cell lymphomas [181] and a camelid antibody directed against the Botulinum neurotoxin, expressed in the chloroplast of *C. reinhardtii,* which prevailed in the gut of mice receiving the molecule by the oral route [182].

Another relevant group of biopharmaceuticals produced in algae is the case of cytokines. Thus far the following cytokines have been targeted: High mobility group protein B1 (HMGB1), Tumor necrosis factor α (TNF-α), Tumor necrosis factor-related apoptosis inducing ligand (TRAIL), Human vascular endothelial growth factor (VEGF), Human interferon β1 (IFN-β1), and IFN-α2a. The former was produced in *D. salina*, while the rest were produced in *C. reinhardtii* [184,185,186]. Nevertheless, this group of biopharmaceuticals has been characterized at very preliminary stages with the exception of IFN-α2a, which showed inhibitory effects on the propagation of the Vesicular stomatitis virus and malignant cells in vivo [187]. The road ahead is still long and glycosylation studies, as well as the in vivo activity, remain to be characterized. 

RNA interference (RNAi) is an effective approach to mediate the degradation of specific mRNAs; including those of viruses [188,189]. One innovative approach recently reported for algae is their use as biofactories and delivery vehicles of functional dsRNA targeting the lethal shrimp yellow head virus via RNA interference, which led to improved survival rates in shrimp fed with the engineered algae [190]. A similar approach was demonstrated to express in microalgae a dsRNA targeting the 3-hydroxykynurenine transaminase (3-HKT), which is critical for the catabolism in mosquitoes [191]. 

## 4. Perspectives 

It is clear that algae biotechnology offers several approaches to generate therapies and vaccines to fight against COVID-19. In regard to the discovery of novel antiviral compounds; this goal implies identification, purification, and characterization of candidates through suitable strain selection and cultivation; followed by downstream biomass processing [192]. The discovery of anti-SARS-CoV-2 agents derived from algae will be accelerated by the exploitation of high-throughput assays to screen such compounds and the selection of the most promising candidates. In this regard, the methods already reported for studying anti-SARS-CoV-1 activities are the immediate paths to be implemented. Interestingly some of these methods are based on GFP expressing replicons, a highly practical approach not requiring the handle of infectious particles [193].

Although the discovery of novel microalgae-based antivirals is a potential field, the already described compounds deserve evaluations to generate solutions in a straightforward manner. The most characterized and promising antiviral compound isolated from algae is in our opinion GRFT given the wide set of studies supporting its activity against enveloped viruses; including SARS-CoV-1 and MERS-CoV. The perspectives for the application of GRFT to fight COVID-19 are crucial since clinical trials could be implemented in the short term as the production system is already established in plants, although implementing its production in recombinant algae is also a possibility. 

**Figure 2:**
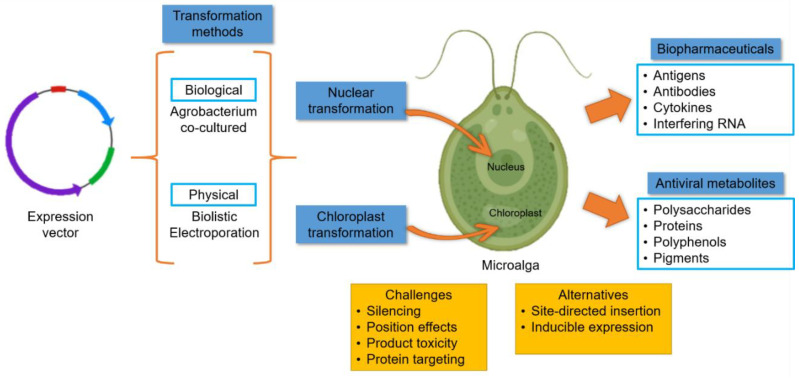
Genetic engineering in algae. Algae can be genetically engineered to improve the production of native antiviral compounds or introduce the biosynthetic pathway for those not produced in algae; moreover, they can be used as biofactories of biopharmaceuticals. The genomes at the nucleus and chloroplast can be engineered with specific genes to achieve the desired trait. The main challenges are genetic instability and low expression often observed in the transformed strains, which can be overcome by using site-directed insertion of the foreign DNA, inducible promoters, and optimized regulatory sequences.

Another key path for this field is to determine the anti-SARS-CoV-2 potential of the already described algae-derived pigments, polysaccharides, and polyphenols assigned as antiviral compounds. Moreover, it should be contemplated that the potential of these applications will in part depend on improving the yields of the target compounds. Changing culture conditions is a strategy that has been followed to enhance the production of desirable metabolites [194]; however, the approach can be further improved by applying genetic engineering (Figure 2). For instance, complete biochemical pathways or multigenetic traits can be introduced via innovative transformation and expression strategies to guarantee genetic stability, protein targeting to specific organelles or secretion, and high expression.

As the production of secondary metabolites involves a complete metabolic pathway, genetic and metabolic engineering can be used to induce the up-regulation or down-regulation of the transcription and translation of key enzymes or to knock-out and knock-in desired genes; as examples that can lead to an efficient production of a target metabolite [195]. 

As an example, the carotenoids biosynthetic pathway has been extensively characterized in algae [14,196,197,198]; therefore, strains capable of yielding native compounds at higher levels or novel compounds can be developed by inactivating or overexpressing endogenous genes or introducing foreign genes [199]. In fact, the carotenoids biosynthesis has been enhanced in *C. reinhardtii* [200,201]. In this regard, increased astaxanthin levels in *H. lacustris* [202], *Chlorella zofingiensis* [203], and *C. reinhardtii* [204] have been achieved. The efficient expression in such approaches was possible by using codon-optimized genes and synthetic promoters that allowed for a strong nuclear gene expression [205,206]. 

The applications of the CRISPR-Cas (clustered regularly interspaced short palindromic repeats–CRISPR associated proteins) 9 system in this field are also pertinent since they could allow suppressing competitive pathways; increasing the production of specific molecules [207,208]. In this sense; RNA interference (RNAi) is another important tool to address this suppression. This type of approaches has been reported for *C. reinhardtii* [209,210] and *D. salina* [211]. Future attempts aimed at engineering the production of secondary metabolites could be based on modifying/introducing the metabolic pathways to direct the metabolic flow into a specific product; combining nuclear and/or chloroplast genetic modification and protein targeting. 

In response to the COVID-19 pandemic; biopharmaceuticals produced in common expression systems (mammalian cells) will be the first approach to cope with the situation. Nonetheless, their use implies as for any platform some limitations such as high production costs and safety concerns related to contamination with mammalian pathogens [212]. The use of algae for producing and even delivering biopharmaceuticals offers an alternative to address the high production cost and cold chain requirements of the products obtained under conventional technologies. 

Overall, the optimized expression systems could be directly applied to the most promising SARS-CoV-2 protective antigens, namely the S protein and its RBD region. The latter is proposed as an antigen able to induce neutralizing antibodies, while the induction of antibodies mediating infection enhancement is avoided. Since these are glycosylated antigens, nuclear expression seems to be the most appropriate approach; although chloroplast-based expression could be explored for RBD, which is simpler than the full-length S protein. Although the assembly of VLPs (virus-like particles) derived from enveloped viruses has not been reported in algae, based on the positive results observed in plants for the case of VLPs from the Influenza virus; one could expect that green algae could lead to a success in this goal. In fact, Medicago (Quebec City, QC, Canada) has announced the production of SARS-CoV-2 VLPs in *N. benthamiana* [213,214]. Exploring distinct signal peptides and specific deletions in the S protein (e.g. deleting the transmembrane domain) are envisaged as important phases to optimize the expression of the S protein in algae. Once expression of the target antigen is achieved, a key aspect will be to implement immunization schemes aimed at inducing robust immune responses in both the systemic compartment and the airways; ensuring both protective effects upon viral challenge and that the antibody dependent enhancement does not occur as consequence of a suboptimal immune response.

The ability of *P. tricornutum* and *C. reinhardtii* to secrete antibodies and enzymes highlights these algae species to produce and secrete glycosylated antigens. For instance, *C. reinhardtii* was engineered to efficiently secrete the ice binding protein (LpIBP), which is a glycoprotein from *Lolium perenne* that is applied as food cryopreservation additive [215]; and the Venus reporter protein, which was expressed with accessory synthetic glycomodules to increase secretion and stability [216]. However, other authors working with *P. tricornutum* have focused on retaining antibodies at the ER to obtain simplified glycosylation patterns that favor their applicability [169]. Another aspect that deserves attention is the difficulty for purifying the recombinant protein secreted to the culture medium due to the presence of cell-wall components (e.g. insoluble (hydroxy)proline-rich glycoproteins). As an alternative to cope with this issue, fusion partners based on the *Lolium perenne* ice binding protein and a fungal hydrophobin tag have been proposed to enhance secretion and facilitate the purification by the application of aqueous two-phase (ATPS) extraction [171]. All these approaches provide a valuable reference to design strategies for the production of SARS-CoV-2 S protein, RBD, and anti-SARS-CoV-2 antibodies. 

Even though nuclear stable expression offers the possibility of producing glycosylated antigens and secreting them to facilitate purification; secretion can be limited by the cell wall and should be evaluated case by case. It is well known that glycosylation influences the safety and efficacy of antigens and antibodies. It is interesting to note that recent experimental and computational evidences for N- and O-glycosylation have led to the design of glyco-engineering approaches in algae [217]. 

All this knowledge offers the perspectives to achieve the production of bioproducts with specific glycan patterns that could ultimately optimize their functionality [217]. In regard to antibodies production, although chloroplast has proven capacity to produce full-length antibodies making it the first line of action; exploring nuclear expression is an opportunity to obtain a product that is glycosylated and exported to the culture medium for simplified purification. In this arena, the race to develop monoclonal antibodies able to serve as therapy for COVID-19 was immediately started; standing as the most rapid approach to develop biopharmaceuticals compared to vaccines [218]. Given the high genetic similarity between SARS-CoV-1 and 2, a SARS-CoV-1 RBD-specific human neutralizing mAb (CR3022) has proven capacity to cross react with the SARS-CoV-2 RBD with high affinity; targeting an epitope not located at the ACE2-binding site [219]. Therefore, the expression of anti-SARS-CoV-1 antibodies showing cross reactivity against SARS-CoV-2 is proposed as an immediate approach to study the viability of the system for producing antibodies. Since SARS-CoV-2 is replicated and secreted in feces, it has been postulated that the fecal-oral transmission deserves attention. Could algae expressing anti-SARS-CoV-2 antibodies applied by the oral route be used as a measure to block virus replication and spreading? In this respect studies on the oral delivery of nanoantibodies are promising, but they are at the initial stage of development [182]. 

The dsRNA expression system proven in *C. reinhardtii* should be applicable to combat SARS-CoV-2 by engineering the alga to produce specific dsRNA targeting this virus; the system could be assessed as an oral therapy to block intestinal replication. In fact RNAi technology has been applied to mediate silencing of coronaviruses with promising results in terms of inhibition of virus replication [220,221]; moreover, specific RNAi to target SARS-CoV-2 has been already proposed [222].

Another challenge, perhaps the biggest for this field consists in improving protein yields and stability of the genetically engineered algae strains. Crucial factors in this respect consist of overcoming low transformation efficiency, positional side effects, and transcriptional/post-transcriptional gene silencing often observed for nuclear expression [209,223]. Such limitations can be overcome by applying the recent advances mentioned in the previous sections; namely the use of efficient promoters, UV mutants, and new selectable markers. With respect to chloroplast transformation, optimized regulatory sequences and selection approaches could be applied to ensure optimal protein yields.

The overall perspectives in the algae-made biopharmaceuticals field also comprise scaling-up the production processes under good manufacturing practices (GMP) and establishing academia-industry relationships, which offer the potential to complete preclinical evaluation and perform clinical trials.

## 5. Conclusions 

With the recent COVID-19 pandemic outbreak, it is urgent to resume coronavirus research to find possible therapeutic agents against SARS-CoV-2; having in mind those with proven activity against SARS-CoV-1 as the starting point [224]. Algae biotechnology has much to offer in the fight against SARS-CoV-2 by serving as source of antiviral compounds and advanced biologicals such as dsRNA, antigens, and antibodies. The development of new genetic engineering tools is progressing and they will allow improvements in terms of recombinant protein yields, secretion, and specific post-translational processing in the algal hosts. The coming months will be critical to evaluate and define the most promising candidates to implement therapeutic and prophylactic approaches against SARS-CoV-2.

## Figures and Tables

**Table 1:** Examples of vaccines produced in innovative expression hosts targeting viral diseases.

Species	Target Pathogen/Antigen	Genetic Engineering Approach	Administration Via and Adjuvant Used	Key Findings	Reference
*C. reinhardtii*	Human papillomavirus A modified version of the E7 oncoprotein	Stable, Chloroplast	s.c./QuilA	Elicited humoral responses and reduced tumor development	[179]
*Schizochytrium* sp.	H1N1 influenza virus Hemagglutinin	Stable, Nuclear	Parenteral/Alone or plus Addavax (squalene-based)	Induced humoral responses and complete immunoprotection upon a pathogen challenge	[180]
*Schizochytrium* sp.	Zika virus Chimeric protein based on the LTB carrier and 3 epitopes from the E protein	Transient	Oral/LTB s.c./Freund’s	The algae-made antigen elicited humoral responses in mice following oral immunization, whose magnitude equals the response induced by s.c. immunization	[183]
